# CD73‐Overexpressing ADSCs Promote Neurogenic Bladder Repair via A2A–JAK1/STAT3‐Mediated Modulation of Inflammation and Macrophage Polarization

**DOI:** 10.1155/sci/8564160

**Published:** 2026-09-29

**Authors:** Guanqun Zhu, Guoyi Jiang, Jiao Huang, Yuxuan He, Zongliang Zhang, Rui Zhang, Xinbao Yin, Kai Zhao, Xiaokun Yang, Woong Jin Bae, Ke Wang

**Affiliations:** ^1^ Department of Urology The Affiliated Hospital of Qingdao University Qingdao Shandong China qdu.edu.cn; ^2^ Catholic Integrative Medicine Research Institute College of Medicine The Catholic University of Korea Seoul Republic of Korea catholic.ac.kr; ^3^ Department of Cardiology Qingdao West Coast New Area Central Hospital Qingdao Shandong China

**Keywords:** adenosine signaling, adipose-derived stem cells, CD73, inflammation, neurogenic bladder

## Abstract

**Background:**

Neurogenic bladder (NB) is characterized by chronic inflammation and bladder tissue injury, and current treatments fail to restore the inflammatory microenvironment or tissue integrity. Although adipose‐derived stem cell (ADSC) therapy shows regenerative potential, its efficacy is limited by inflammation; whether CD73 overexpression enhances ADSC therapeutic effects in NB remains unclear.

**Methods:**

A rat model of NB was established via pelvic nerve (PN) injury and treated with CD73‐overexpressing ADSCs (CD73^+^ADSCs). Bladder function, histological remodeling, and apoptosis were evaluated in vivo. In vitro, conditioned medium (CM) from CD73^+^ADSCs was applied to LPS‐injured rat bladder transitional epithelial cells (RB‐TECs) and macrophages to assess proliferation, migration, apoptosis, inflammatory responses, and macrophage polarization. Key signaling pathways were examined, and label‐free quantitative (LFQ) proteomics was performed to characterize global molecular reprogramming.

**Results:**

CD73^+^ADSCs significantly improved urodynamic function, restored smooth muscle architecture, and reduced apoptosis in NB rats. In vitro, CD73^+^ADSC‐CM promoted urothelial cell proliferation and migration while attenuating cell death. Mechanistically, CD73^+^ADSCs enhanced extracellular adenosine production, at least partly dependent on pharmacologically sensitive A2A receptor signaling, suppressed NF‐κB activation, reduced proinflammatory cytokine release, promoted macrophage polarization toward an M2 phenotype, and inhibited NLRP3 inflammasome activation. Proteomic analysis revealed a global shift toward antiinflammatory and reparative molecular programs.

**Conclusion:**

Our findings suggest that CD73 contributes to the immunomodulatory and reparative properties of ADSCs under inflammatory conditions. By coordinating adenosinergic signaling, inflammatory suppression, and repair‐associated pathways, CD73^+^ADSCs effectively reprogram the NB microenvironment and represent a promising regenerative therapeutic strategy for NB.

## 1. Introduction

Neurogenic bladder (NB) is a common and debilitating complication of neurological disorders, including spinal cord injury, pelvic nerve (PN) injury, multiple sclerosis, and diabetic neuropathy [1]. NB is characterized by impaired bladder sensation, detrusor dysfunction, chronic urinary retention, and progressive structural remodeling of the bladder wall [2]. Over time, persistent inflammation, apoptosis of urothelial and smooth muscle cells, and excessive fibrosis contribute to a decline in bladder compliance and eventual renal deterioration [3]. Current clinical management relies primarily on catheterization, anticholinergic agents, or botulinum toxin injections, all of which offer symptomatic relief but fail to address the underlying pathological microenvironment or promote lasting tissue repair [4]. Therefore, the development of regenerative strategies capable of restoring the bladder structure and function is urgently needed.

Adipose‐derived stem cells (ADSCs) are an attractive candidate for bladder regeneration due to their accessibility, multipotency, paracrine activity, and immunomodulatory properties [5]. ADSCs have been shown to ameliorate tissue injury in a variety of models by secreting trophic factors that support cell survival, attenuate inflammation, and enhance angiogenesis [6]. However, several challenges limit their therapeutic efficacy, including the hostile inflammatory microenvironment of NB and insufficient activation of reparative signaling pathways within stem cells and recipient tissues. Thus, strategies that enhance the biological potency of ADSCs may significantly improve their regenerative capacity.

CD73 (ecto‐5^′^‐nucleotidase), a glycosylphosphatidylinositol‐anchored enzyme highly expressed on mesenchymal stem cells, catalyzes the conversion of extracellular AMP to adenosine—a key immunoregulatory and tissue‐protective molecule [7]. Through adenosine A2A receptor activation, CD73 modulates critical signaling pathways involved in cell proliferation, inflammation, apoptosis, and immune polarization [8]. Increasing evidence suggests that CD73 plays an essential role in tissue repair in organs [9, 10]; however, its therapeutic potential in NB has not been fully explored. Furthermore, whether CD73 overexpression can potentiate the reparative functions of ADSCs through coordinated regulation of inflammatory signaling and cellular responses remains unknown.

Among the downstream pathways of CD73–adenosine signaling, the JAK1/STAT3 axis is of particular interest [11]. STAT3 is a master regulator of cell survival, proliferation, and tissue regeneration, and its activation has been associated with enhanced wound healing and antiinflammatory responses [12]. Conversely, NF‐κB, a well‐established mediator of inflammatory injury, drives cytokine production, urothelial damage, and fibrotic remodeling in bladder disorders [13]. The interplay between STAT3 activation and NF‐κB suppression represents a critical regulatory checkpoint for restoring epithelial homeostasis and promoting tissue repair. Whether CD73‐overexpressing ADSCs (CD73^+^ADSCs) orchestrate these interconnected pathways to generate a more favorable environment for bladder regeneration remains unclear.

Against this background, the present study was designed to evaluate the therapeutic effects of CD73^+^ADSCs in a rat model of NB and to clarify the molecular pathways potentially involved in this process. We focused on whether CD73^+^ADSCs may influence adenosine A2A receptor‐related JAK1/STAT3 signaling, NF‐κB‐mediated inflammatory responses, macrophage polarization, and NLRP3 inflammasome activation during NB repair. To further characterize CD73‐associated molecular changes, integrated proteomic analysis was also conducted. We hypothesized that CD73 overexpression may enhance the reparative and immunomodulatory properties of ADSCs in the NB microenvironment.

## 2. Materials and Methods

### 2.1. Generation and Characterization of Genetically Modified ADSCs

Adipose‐derived mesenchymal stem cells (ADSCs) were purchased from a certified commercial supplier (Procell, Wuhan, Hubei, China) and cultured in a rat ADSC‐specific medium (Procell, Wuhan, Hubei, China) under standard conditions (37°C, 5% CO_2_, and saturated humidity). Before genetic manipulation, ADSCs were characterized on the basis of their spindle‐shaped morphology, growth characteristics, and expression of canonical mesenchymal stem cell surface markers to ensure their suitability for subsequent experiments.

To generate the CD73‐overexpressing model, an optimized coding sequence of rat CD73 (NC_000012.12) was synthesized and cloned into an expression vector, followed by sequence confirmation (OBIO, Nanjing, China). ADSCs were seeded into 12‐well plates at a density of 5 × 10^5^ cells per well and transfected according to the manufacturer’s instructions once the cells had reached a stable condition. ADSCs transfected with the empty vector were used as negative controls. At 48 h after transfection, the transfection efficiency was evaluated by flow cytometry, and CD73 mRNA and protein expression levels were assessed by RT‐qPCR and Western blotting, respectively. The transfected cells were subsequently screened, and CD73^+^ADSCs with stable expression and favorable biological characteristics were selected for further experiments.

To establish the CD73‐knockdown model, ADSCs were transfected with CD73‐targeting small interfering RNA (siRNA) using Lipofectamine 3000 (Thermo Fisher Scientific, Rockford, IL, USA) in accordance with the manufacturer’s instructions. The siRNA target sequences are provided in the Supporting Information. Cells treated with scrambled siRNA or the corresponding negative control were used as controls. At 48 h after transfection, the CD73‐knockdown efficiency was evaluated by Western blotting (Figure S2). In addition, enzymatic activity assays were performed to further confirm the functional suppression of CD73.

Ultimately, three ADSC populations were generated and used for subsequent analyses: CD73‐deficient ADSCs (CD73^−^ADSCs), empty vector‐transfected ADSCs (CD73^ev^ADSCs), and CD73^+^ADSCs. Before their use in in vitro and in vivo functional experiments, all cell groups were reevaluated in terms of morphological characteristics, cell viability, proliferative capacity, expression of canonical ADSC surface markers, CD73 protein expression, and enzymatic activity. In addition, cell quality was monitored both before and after cryopreservation, and only cell preparations with postthaw viability ≥ 95% and stable CD73 expression were used in subsequent experiments to ensure the reliability and reproducibility of the study.

For conditioned medium (CM) preparation, CD73^−^ADSCs, CD73^ev^ADSCs, and CD73^+^ADSCs were plated in 100‐mm culture dishes. Once cells reached full adherence, the culture medium was aspirated, and cells were washed three times with PBS. A serum‐free basal medium was then added, and cells were incubated for an additional 24 h under standard culture conditions. The collected supernatants were clarified by centrifugation at 3000 rpm for 15 min at 4°C and stored at −80°C until further use.

### 2.2. Cell Culture in CM

The rat bladder transitional epithelial cell (RB‐TECs, Procell, Wuhan, Hubei, China) line was exposed to CM for 48 h. Cells were assigned to eight treatment groups: untreated control; lipopolysaccharide (LPS, 1 μg/mL); LPS combined with CM from CD73^−^ADSCs; LPS combined with CM from CD73^ev^ADSCs; LPS combined with CM from CD73^+^ADSCs; LPS plus CD73^+^ADSC‐CM in the presence of the adenosine A2A receptor antagonist ZM241385; LPS plus CD73^+^ADSC‐CM with the JAK1 inhibitor NS‐018; and LPS plus CD73^+^ADSC‐CM with the STAT3 inhibitor S3I‐201. Following 48 h of treatment, both cells and culture supernatants were harvested for subsequent analyses.

RAW264.7 macrophages were cultured in CM for 48 h under identical conditions. Five experimental groups were included: control, LPS, LPS + CD73^+^ADSCs, LPS + CD73^+^ADSCs + adenosine A2A receptor antagonist ZM241385, and LPS + CD73^+^ADSCs + JAK1 inhibitor NS‐018. Cells and supernatants were harvested for subsequent assays. Detailed information on all inhibitors (including suppliers, catalog numbers, and working concentrations) is provided in the Supporting Information.

### 2.3. Cell Proliferation and Migration Assays

Cell proliferation of RB‐TECs treated with CM was assessed using the Cell Counting Kit‐8 (CCK‐8; MCE, Shanghai, China). Cells were plated in 96‐well plates at a density of 2 × 103 cells per well, with three technical replicates for each condition, and cultured in CM obtained from the control, LPS, LPS + CD73^−^ADSC, LPS + CD73^ev^ADSC, or LPS + CD73^+^ADSC groups. Cell viability was evaluated at 0, 24, 48, 72, and 96 h after treatment. At each time point, the CCK‐8 reagent was added to the wells and incubated for 2 h at 37°C. Absorbance was subsequently recorded at 450 nm using a microplate reader (Safire2, Tecan, Männedorf, Switzerland). Proliferation curves were constructed based on the averaged absorbance values from the replicate wells.

Cell migration of RB‐TECs was assessed using a wound healing assay. RB‐TECs were treated with CM obtained from control, LPS, LPS + CD73^−^ADSCs, LPS + CD73^ev^ADSCs, and LPS + CD73^+^ADSCs, and migration was quantified after 24 h of incubation. All experiments were performed in triplicate.

### 2.4. Experimental Animals and Study Design

Eight‐week‐old male Sprague–Dawley rats weighing 250–300 g were obtained from an accredited supplier (Pengyue, Jinan, Shandong, China). Animals were randomly assigned to five experimental groups (*n* = 8 per group): NB, NB treated with CD73^−^ADSCs, NB treated with CD73^ev^ADSCs, NB treated with CD73^+^ADSCs, and a sham‐operated control group. Following the induction of anesthesia, the major pelvic ganglion (MPG) and bilateral PNs were surgically exposed through a lower midline abdominal incision. In NB model groups, NB was established by combined injury to the MPG and transection of both PNs, in accordance with previously described methods [14]. Sham‐operated animals underwent identical surgical exposure without nerve damage. Postoperatively, rats were housed individually under standard laboratory conditions with unrestricted access to food and water.

### 2.5. Intratissue Injection of ADSCs

Two weeks following successful induction of the NB model, rats assigned to the NB + CD73^−^ADSC, NB + CD73^ev^ADSC, and NB + CD73^+^ADSC groups were administered intramural bladder injections of the corresponding ADSC populations. Under general anesthesia, cells were injected into four evenly distributed sites of the bladder wall (at the 3, 6, 9, and 12 o’clock positions). Each animal received a total of 1 × 10^6^ ADSCs suspended in PBS, following previously established protocols [15]. Rats in the NB‐ and sham‐operated groups were injected with an equivalent volume of PBS alone. For in vivo cell tracking, ADSCs were fluorescently labeled prior to transplantation using CellTrackerTM CM‐DiI (MCE, Shanghai, China) in accordance with the manufacturer’s instructions.

### 2.6. Voiding Spot Assay (VSA)

VSA was performed to evaluate urinary function in rats. Briefly, animals were individually housed in metabolic cages, and filter paper was placed beneath the wire‐mesh floor to collect urine over a 3 h period. After collection, the filter papers were photographed under ultraviolet illumination. Urine spot images were analyzed using ImageJ software (NIH, MD, USA) to quantify the area of individual voiding spots. The number of urine spots was defined as the voiding frequency. Based on prior calibration, a urine volume of 50 μL corresponded to a spot area of ~2.8 cm^2^ on the filter paper. VSA was conducted 4 weeks after the treatment. All measurements and data analyses were performed in a blinded manner to minimize potential bias related to group allocation and treatment.

### 2.7. Cystometry Test

Cystometric evaluation was carried out in all groups 4 weeks after ADSC transplantation. The procedure was performed according to established protocols with minor adjustments [16]. Briefly, rats were anesthetized, and a lower midline abdominal incision was made to expose the urinary bladder. A 25‐gauge needle connected to polyethylene tubing was inserted into the bladder dome and fixed in position. After complete bladder evacuation, warmed saline was continuously infused at a rate of 0.4 mL/min to induce repeated micturition cycles. Intravesical pressure was continuously recorded using a multichannel polygraph system (Grass 7D, Grass Institute Co., Quincy, MA, USA). Key cystometric parameters, including the intercontraction interval and maximum voiding pressure, were analyzed. Upon completion of cystometric measurements, bladder tissues were harvested for subsequent histological and molecular studies.

### 2.8. Immunofluorescence Staining and Histology

Immunofluorescence staining was performed to evaluate the expression of cytokines and related markers. Bladder tissues were fixed in 4% paraformaldehyde at 4°C for 24 h. After routine permeabilization and blocking, samples were incubated with the appropriate primary antibodies (listed in the Supporting Information), followed by incubation with corresponding fluorescently labeled secondary antibodies. Images were acquired using a confocal laser scanning microscope (Leica TCS SP8, Leica Microsystems, Wetzlar, Germany), and fluorescence intensity was quantitatively analyzed using NIS‐Elements Viewer software (Nikon, Tokyo, Japan).

Immunohistochemical staining was performed to assess inflammatory changes in the bladder tissue. Paraffin‐embedded bladder sections were processed and stained using commercially available immunohistochemistry kits according to the manufacturer’s instructions, with the primary antibodies listed in the Supporting Information. Bright‐field images were acquired using a light microscope (Nikon Ts2R, Nikon, Tokyo, Japan), and mean staining intensity was quantified with NIS‐Elements Viewer software.

Histopathological alterations following stem cell therapy were evaluated using hematoxylin and eosin (HE) staining. Bladder tissues from each experimental group were processed and stained with a commercial HE staining kit (Solarbio, Beijing, China) in accordance with the manufacturer’s instructions.

Collagen accumulation and smooth muscle organization were evaluated using Masson’s trichrome staining. Tissue sections from all experimental groups were processed simultaneously with a commercial Masson staining kit (Solarbio, Beijing, China) to reduce interbatch variability. Representative images were captured using a Nikon Ts2R microscope (Nikon, Tokyo, Japan), and staining intensity was quantitatively analyzed with NIS‐Elements Viewer software. At least three independent samples were examined for each group.

### 2.9. Western Blot Analysis

Bladder tissues and cultured cells were lysed on ice using RIPA buffer supplemented with phosphatase inhibitors and an EDTA‐free protease inhibitor cocktail (Elabscience, Wuhan, Hubei, China). The resulting lysates were clarified by centrifugation at 15,000 ×*g* for 15 min at 4°C, and protein concentrations in the supernatants were quantified using a BCA. Equivalent amounts of total protein were resolved by SDS‐polyacrylamide gel electrophoresis and subsequently transferred onto membranes. The membranes were incubated with specific primary antibodies (detailed in the Supporting Information), followed by incubation with corresponding horseradish peroxidase‐conjugated secondary antibodies. Immunoreactive signals were visualized using a chemiluminescence detection system. Densitometric analysis of protein bands was performed using ImageJ software (NIH, Bethesda, MD, USA).

### 2.10. Enzyme‐Linked Immunosorbent Assay (ELISA)

The levels of pro‐ and antiinflammatory cytokines, including IL‐1β, IL‐4, IL‐6, IL‐10, TNF‐α, and IL‐18, were quantified in bladder tissues following ADSC treatment as well as in cells cultured with CM using an ELISA. Cytokine concentrations were measured with commercially available ELISA kits (BioLegend, San Diego, CA, USA) following the manufacturers protocols. Optical density was measured at 450 nm using a microplate reader (Safire2, Tecan, Männedorf, Switzerland). All assays were conducted with at least three independent biological replicates.

### 2.11. Flow Cytometry

Cell apoptosis was quantified using an Annexin V–FITC/propidium iodide (PI) apoptosis detection kit (Elabscience, Wuhan, Hubei, China) in accordance with the manufacturer’s instructions. Following staining, the samples were immediately analyzed using a NovoCyte flow cytometer (ACEA Biosciences, CA, USA). To evaluate macrophage polarization, RAW264.7 cells cultured with CM were collected and washed with PBS. For intracellular marker staining, cells were blocked with 5% bovine serum albumin and incubated for 30 min at room temperature with FITC‐conjugated antimouse CD86 (Elabscience, Wuhan, Hubei, China; 1:100 dilution) and PE‐conjugated antimouse CD163 antibodies (Elabscience, Wuhan, Hubei, China; 1:100 dilution). After washing, the cells were analyzed by flow cytometry. The percentages of CD86‐positive and CD163‐positive cells were used to represent M1 and M2 macrophage phenotypes, respectively.

### 2.12. TUNEL Assay

Apoptotic cell death in bladder tissues was assessed by terminal deoxynucleotidyl transferase‐mediated dUTP nick‐end labeling (TUNEL) staining. Excised bladder specimens were fixed in 4% paraformaldehyde at 4°C for 24 h and subsequently paraffin‐embedded. Tissue sections were processed using a commercial TUNEL assay kit (Elabscience, Wuhan, Hubei, China) in accordance with the manufacturer’s protocol. Following incubation with the TUNEL reaction mixture, the cell nuclei were counterstained with DAPI. Fluorescence images were captured using a confocal laser scanning microscope (Leica TCS SP8, Leica Microsystems, Wetzlar, Germany), and TUNEL‐positive signals were quantitatively analyzed with NIS‐Elements Viewer software (Nikon, Tokyo, Japan). At least three independent samples were analyzed for each group.

### 2.13. Proteomics

SV‐HUC‐1 cells (Procell, Wuhan, Hubei, China) were harvested after 48 h of treatment, snap‐frozen in liquid nitrogen, and lysed following TCA/acetone precipitation. Protein pellets were resuspended, denatured, sonicated, and clarified by centrifugation, and protein concentrations were determined using a BCA assay. For label‐free quantitative (LFQ) proteomics, 100 μg of protein per sample was reduced with TCEP, alkylated with chloroacetamide, and digested overnight with sequencing‐grade trypsin. Peptides were desalted, dried, and reconstituted in 0.1% formic acid.

Peptide analysis was performed using a nanoElute UHPLC system coupled to a TimsTOF Pro mass spectrometer. Separation was achieved on a 25 cm × 75 μm C18 column at 50°C with a flow rate of 300 nL/min. Protein identification and label‐free quantification were conducted at a 1% false discovery rate at both the peptide and protein levels.

### 2.14. Statistical Analysis

Statistical analyses were performed using SPSS software (Version 22.0; IBM, Armonk, NY, USA) and the R statistical computing environment (Version 4.4.1; R Foundation for Statistical Computing, Vienna, Austria). Quantitative data are presented as the mean ± standard deviation (SD). Unless otherwise specified, in vitro experiments were performed using at least three independent biological replicates, with each biological replicate measured in triplicate as technical replicates, and the averaged value from each biological replicate was used for statistical analysis.

Prior to hypothesis testing, data distribution was assessed using the Shapiro–Wilk test for normality, and the homogeneity of variance was evaluated using Levene’s test. Sample sizes were estimated based on an a priori power analysis to achieve 80% statistical power at a two‐sided *α* level of 0.05. For comparisons among more than two groups, one‐way analysis of variance (ANOVA) was used, followed by Tukey’s multiple‐comparison test when equal variances were assumed. When variance homogeneity was not met, Welch’s ANOVA, followed by an appropriate post‐hoc test, was applied. Comparisons between the two groups were conducted using an unpaired two‐tailed Student’s *t*‐test. For data that did not satisfy the assumptions of parametric testing, the Mann–Whitney *U* test or Kruskal–Wallis test was used, as appropriate.

For histological, immunohistochemical, and immunofluorescence quantification, measurements were obtained from three randomly selected nonoverlapping fields per section/sample and averaged to generate one value for each biological replicate. Western blot densitometry, RT‐qPCR, and other quantitative assays were analyzed from independent experiments, as described above. The exact sample size, replicate type, and statistical test used for each dataset are provided in the corresponding figure legends. A *p*‐value < 0.05 was considered statistically significant.

## 3. Results

### 3.1. CD73^+^ADSCs Enhance Bladder Structural Repair and Improve Urodynamic Function in NB Rats

To demonstrate the role of CD73^+^ADSCs in improving NB function and promoting bladder tissue repair, a NB animal model was established, and the effects of CD73^+^ADSCs on voiding function, structural remodeling, apoptosis, and the expression of related signaling molecules were systematically evaluated.

Cystometric analysis demonstrated severe bladder dysfunction in the NB group, characterized by irregular voiding patterns and reduced bladder compliance compared with sham controls (Figure 1A). Quantitative analysis revealed that NB rats exhibited significantly increased maximum bladder pressure and decreased bladder capacity, both of which were markedly improved following CD73^+^ADSC transplantation (Figure 1B,D). Histological examination using HE staining revealed extensive structural disruption and inflammatory infiltration in NB bladder tissues, whereas CD73^+^ADSC treatment substantially alleviated these pathological changes (Figure 1C). Masson’s trichrome staining further showed excessive collagen deposition and smooth muscle disorganization in NB bladders, which were significantly reduced following CD73^+^ADSC administration, indicating restoration of smooth muscle integrity (Figure 1E,F). CM‐DiI tracking demonstrated that ADSCs successfully migrated to and engrafted within bladder tissues, with significantly stronger fluorescence signals observed in the CD73^+^ADSC group compared with the control ADSCs group (Figure 1G,H). TUNEL staining demonstrated a marked increase in apoptotic cells in NB bladder tissues compared with sham controls, whereas CD73^+^ADSC treatment significantly reduced apoptotic cell numbers (Figure 1I,J). VSA results indicated that CD73^+^ADSCs markedly improved voiding patterns in rats, with the urine spot number and distribution approaching those of the Sham group (Figure 1K,L). Western blot analysis and quantitative assessment further confirmed that CD73^+^ADSCs significantly increased CD73 protein expression and A2A in bladder tissues (Figure 1M,N and Figure S1).

Figure 1CD73‐overexpressing ADSCs enhance bladder structural repair and improve urodynamic function in neurogenic bladder rats. (A) Representative cystometry results for each group in vivo. (B, D) Quantitative analysis of cystometry results, including maximal bladder pressure and intercontraction interval (C) HE staining of bladder tissues in vivo, scale bar = 100 μm. (E) Masson staining of bladder tissues in vivo, scale bar = 100 μm. (F) Quantitative analysis of smooth muscle content in the bladder (yellow square). (G) CM‐DiI‐labeled in bladder tissues of each group in vivo, scale bar = 100 μm. (H) Quantification of ADSCs in bladder tissues. (I) TUNEL staining of bladder tissues in vivo, scale bar = 100 μm. (J) Quantification of apoptotic cells in bladder tissues. (K) Results of the voiding spot assay (VSA) in different groups of rats. (L) Quantification of VSA. (M) Western blot analysis of CD73 expression in rat bladder tissues. (N) Quantitative analysis of CD73 and A2A expression. Statistical significance is indicated as  ^∗^ *p* < 0.05,  ^∗∗^ *p* < 0.01, and  ^∗∗∗^ *p* < 0.001. All experiments were independently performed in triplicate. The full‐length, uncropped Western blot images are provided in Figure S1.

**Figure:**
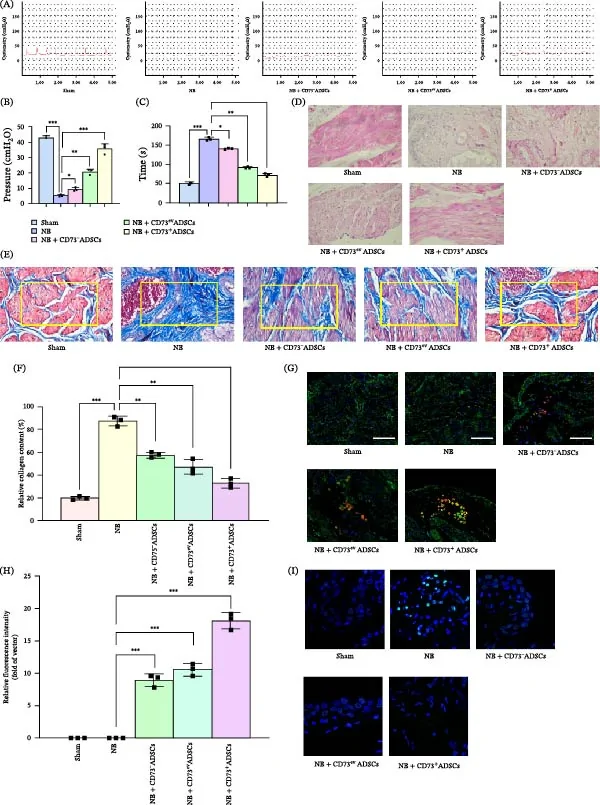

**Figure:**
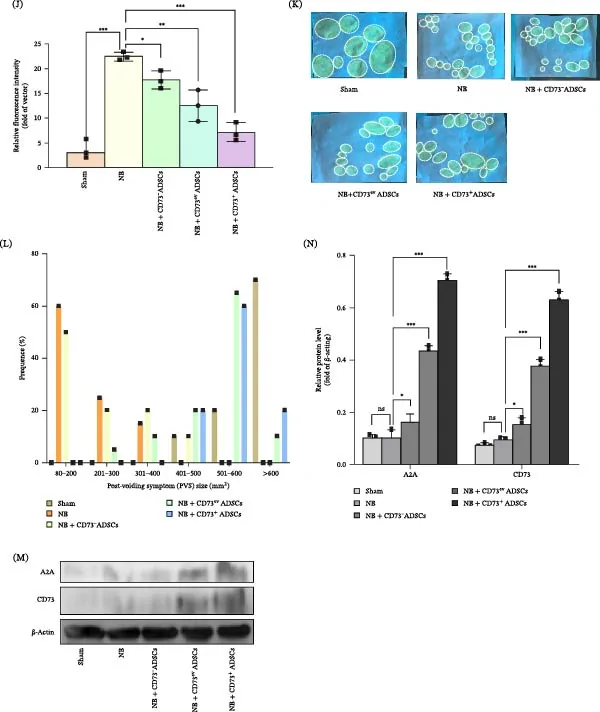

Collectively, these findings demonstrate that CD73^+^ADSCs significantly improve voiding function in NB rats, promote bladder tissue repair, increase the smooth muscle content, and reduce fibrosis. In addition, CD73^+^ADSCs effectively suppress apoptosis in both bladder tissues and cellular models, accompanied by significant upregulation of CD73 and A2A receptor expression in bladder tissues. These results suggest that CD73^+^ADSCs exert a pronounced protective effect in NB injury and may mediate their therapeutic benefits through the modulation of related signaling pathways.

### 3.2. CD73^+^ADSCs Induce Global Proteomic Reprogramming in the LPS‐Induced Microenvironment

To comprehensively characterize the molecular mechanisms underlying the immunomodulatory effects of CD73^+^ADSCs, LFQ proteomic profiling was performed in cells exposed to LPS with or without CD73^+^ADSC treatment.

As shown in Figure 2A, substantial overlap was observed between the proteins identified in the LPS and LPS + CD73^+^ groups, with 2785 shared proteins, while 193 and 512 proteins were uniquely detected in the LPS and LPS + CD73^+^ groups, respectively, indicating marked proteomic remodeling following CD73^+^ADSC intervention. Differential expression analysis based on LFQ‐derived fold changes revealed a discrete subset of significantly regulated proteins (Figure 2B). Notably, CD73^+^ADSC treatment resulted in the predominant downregulation of inflammation‐associated proteins, including key regulators of the NF‐κB signaling pathway, such as NFKB1 and NFKB2, suggesting effective suppression of proinflammatory signaling at the proteomic level. Hierarchical clustering of differentially expressed proteins further demonstrated distinct expression patterns between the LPS and LPS + CD73^+^ groups (Figure 2C), with coordinated downregulation of immune‐ and stress‐related proteins and concomitant upregulation of proteins associated with cellular metabolism and homeostasis, supporting a broad reprogramming of the inflammatory proteome. Functional enrichment analysis revealed significant alterations across multiple biological processes and pathways. The circular functional enrichment analysis further summarized the major significantly enriched pathways and integrated the proportions of upregulated and downregulated proteins, fold enrichment, and statistical significance (Figure 2D). This analysis revealed coordinated pathway‐level remodeling rather than isolated changes in individual proteins, further supporting the broad regulatory effect of CD73^+^ADSCs on inflammation‐, metabolism‐, and cellular homeostasis‐related biological processes. KEGG pathway analysis demonstrated a pronounced negative enrichment of the lysosome pathway toward the LPS group (Figure 2E), indicating attenuation of lysosome‐associated inflammatory and stress‐response programs following CD73^+^ADSC treatment. In parallel, adipocytokine signaling, a pathway closely linked to inflammation‐associated immunometabolic regulation, also exhibited significant negative enrichment in the LPS + CD73^+^ group (Figure 2F), further supporting the suppression of inflammatory metabolic signaling. In contrast, DNA replication‐related pathways were positively enriched in the LPS + CD73^+^ group (Figure 2G), reflecting activation of replication‐associated and proliferative programs, which are indicative of enhanced cellular repair and regenerative potential following CD73^+^ADSC intervention.

**Figure 2:**
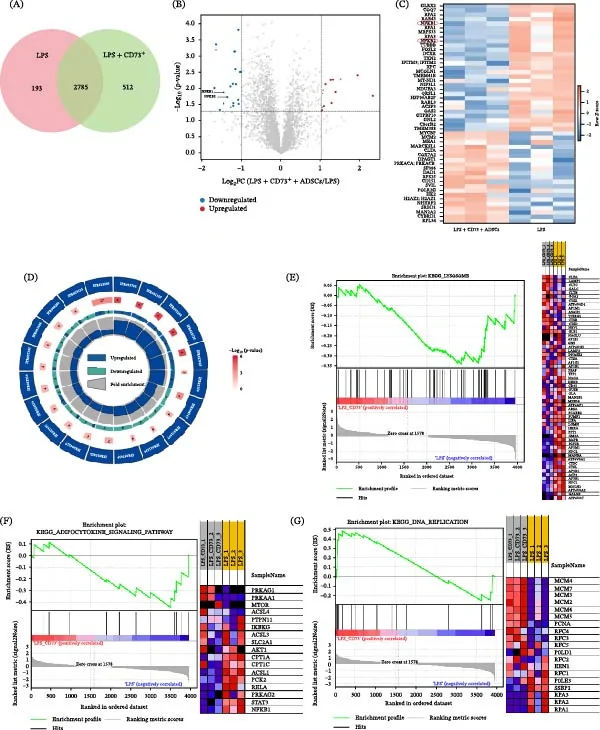
CD73^+^ADSCs induce global proteomic reprogramming in the LPS‐induced microenvironment. (A) Overlap of proteins identified by label‐free quantitative proteomics in the LPS and LPS + CD73^+^ADSC groups. (B) Volcano plot showing differentially expressed proteins between the LPS and LPS + CD73^+^ADSC groups. (C) Heatmap of selected differentially expressed proteins across experimental groups. (D) Functional enrichment analysis of differentially expressed proteins. (E) Gene set enrichment analysis (GSEA) of the KEGG lysosome pathway. (F) GSEA of the KEGG adipocytokine signaling pathway. (G) GSEA of the KEGG DNA replication pathway. Proteomic analysis was performed using label‐free quantification.

Collectively, these proteomic data demonstrate that CD73^+^ADSCs induce a global remodeling of the NB inflammatory proteome, characterized by the suppression of inflammatory and immunometabolic pathways and concurrent activation of molecular programs associated with cellular repair and proliferation under LPS stimulation.

### 3.3. CD73 Promotes Proliferation, Migration, and Survival of NB‐Injured RB‐TECs in Association With the A2A–JAK1/STAT3 Signaling Pathway

To further investigate the molecular mechanisms by which CD73^+^ADSCs promote repair in NB, their effects on RB‐TECs proliferation, migration, apoptosis, and activation of related signaling pathways were evaluated.

In vitro wound healing assays demonstrated that NB injury markedly impaired RB‐TECs migratory capacity, whereas treatment with CD73^+^ADSCs significantly accelerated wound closure compared with the LPS group (Figure 3A, B). Flow cytometric analysis further revealed a pronounced increase in apoptotic cells following LPS injury, which was substantially attenuated by CD73^+^ADSC treatment, indicating enhanced cell survival (Figure 3C). To investigate the underlying signaling mechanisms in vivo, immunofluorescence staining of bladder tissues revealed significantly increased phosphorylation of JAK1 and STAT3 in the CD73^+^ADSC‐treated group compared with the NB and control groups (Figure 3D, E), while ELISA demonstrated that CD73^+^ADSCs significantly increased extracellular adenosine levels (Figure S1), further supporting involvement of the CD73–adenosine‐associated signaling axis. Consistently, western blot analysis confirmed robust activation of the JAK1/STAT3 pathway, as evidenced by elevated *p*‐JAK1 and *p*‐STAT3 levels, accompanied by a concomitant reduction in NF‐κB phosphorylation following CD73^+^ADSC treatment (Figure 3F, G, Figure S2). In parallel, CCK‐8 assays demonstrated that CD73^+^ADSCs significantly enhanced RB‐TECs proliferative capacity under LPS conditions, further supporting a prorepair role of CD73‐mediated signaling (Figure 3H).

**Figure 3:**
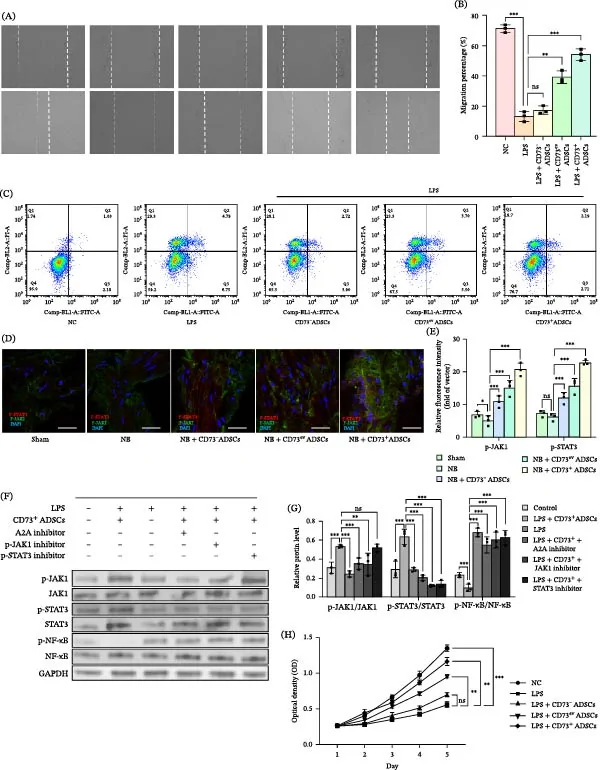
CD73 promotes proliferation, migration, and survival of NB‐injured urothelial cells in association with the A2A–JAK1/STAT3 signaling pathway. (A) Wound healing assay of each group in vitro, original magnification ×4. (B) Quantitative analysis of wound healing assay. (C) Flow cytometry analysis of apoptosis in cultured cells of each group in vitro. (D) Immunofluorescence staining of *p*‐JAK1 and *p*‐STAT3 in bladder tissues in vivo, scale bar = 25 μm. (E) Quantitative analysis of immunofluorescence results for *p*‐JAK1 and *p*‐STAT3 in rat bladder tissues. (F) Western blot results of *p*‐JAK1, JAK1, *p*‐STAT3, STAT3, *p*‐NF‐κB, and NF‐κB of each group in vitro. (G) Quantitative analysis of *p*‐JAK1, JAK1, *p*‐STAT3, STAT3, *p*‐NF‐κB, and NF‐κB expression in vitro. (H) CCK‐8 assay of each group in vitro. Statistical significance is indicated as  ^∗^ *p* < 0.05,  ^∗∗^ *p* < 0.01, and  ^∗∗∗^ *p* < 0.001. All experiments were independently performed in triplicate. All experiments were independently performed in triplicate. The full‐length, uncropped Western blot images are provided in Figure S2. CCK‐8 data are presented in Table S9.

These findings together suggest that CD73^+^ADSCs markedly promote the proliferation and migration of RB‐TECs while effectively inhibiting apoptosis. These effects are accompanied by significant activation of the A2A–JAK1/STAT3 signaling pathway and suppression of NF‐κB signaling, suggesting that CD73^+^ADSCs play a critical regulatory role in urothelial repair through the activation of the A2A–JAK1/STAT3 axis.

### 3.4. CD73^+^ADSCs Attenuate Inflammatory Responses and Suppress NF‐κB Activation in the NB Microenvironment

To determine whether CD73^+^ADSCs enhance the antiinflammatory capacity of the NB microenvironment, inflammatory responses were systematically evaluated at both the cytokine and signaling levels.

As shown in Figure 4A, the establishment of the in vitro NB microenvironment model resulted in a significant increase in the levels of proinflammatory cytokines, including IL‐1β, IL‐6, and TNF‐α, compared with the control group, while antiinflammatory cytokines were relatively reduced. Treatment with CD73^+^ADSCs markedly attenuated this inflammatory response, as evidenced by a significant decrease in proinflammatory cytokine secretion and a concomitant increase in antiinflammatory cytokines (IL‐4 and IL‐10). Notably, inhibition of A2A signaling partially reversed these effects, indicating that the immunomodulatory function of CD73^+^ADSCs is at least partially A2A receptor‐dependent.

**Figure 4:**
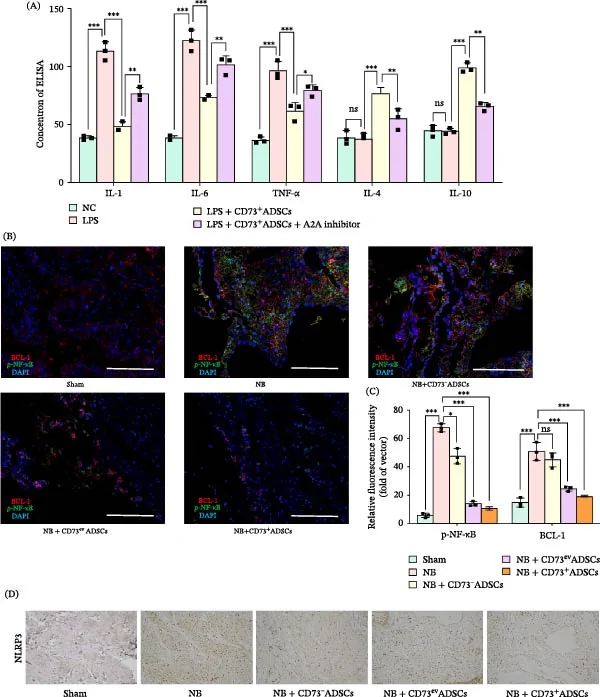
CD73‐overexpressing ADSCs attenuate inflammatory responses and suppress NF‐κB activation in the NB microenvironment. (A) ELISA analysis of TNF‐α, IL‐1β, IL‐4, IL‐6, and IL‐10 levels in each group in vitro. (B) Immunofluorescence staining of BCL‐1 and *p*‐NF‐κB expression in vitro, scale bar = 25 μm. (C) Quantitative analysis of BCL‐1 and *p*‐NF‐κB expression in vitro. (D) Immunohistochemical staining of NLRP3 in bladder tissues in vivo (scale bar = 100 μm). Statistical significance is indicated as  ^∗^ *p* < 0.05,  ^∗∗^ *p* < 0.01, and  ^∗∗∗^ *p* < 0.001. All experiments were independently performed in triplicate. ELISA data are summarized in Table S2–S6.

Immunofluorescence staining further demonstrated that NB injury induced robust activation of NF‐κB signaling, whereas treatment with CD73^+^ADSCs significantly suppressed *p*‐NF‐κB expression and reduced inflammatory signaling activity (Figure 4B, C). Quantitative analysis confirmed a marked reduction in NF‐κB activation following CD73^+^ADSC treatment compared with the NB group. Moreover, immunohistochemical analysis revealed that NLRP3 expression was substantially upregulated in the NB group, while CD73^+^ADSC treatment significantly reduced NLRP3 levels (Figure 4D), suggesting inhibition of inflammasome activation and further attenuation of the inflammatory microenvironment.

Collectively, these results demonstrate that CD73^+^ADSCs effectively suppress inflammatory responses within the NB microenvironment, reduce proinflammatory cytokine production, inhibit NF‐κB signaling activation, and downregulate NLRP3 inflammasome expression. These findings indicate that CD73^+^ADSCs exert a pronounced antiinflammatory effect and contribute to the restoration of an antiinflammatory microenvironment during NB injury repair.

### 3.5. CD73^+^ADSCs Regulate Macrophage Polarization Toward the M2 and Inhibit NLRP3 Inflammasome Activation

After confirming that CD73^+^ADSCs markedly suppress inflammatory responses and downregulate NF‐κB signaling activation in the NB microenvironment, their effects on key inflammatory regulatory processes, including macrophage polarization and NLRP3 inflammasome activation, were further investigated.

To investigate whether the antiinflammatory effects of CD73^+^ADSCs are associated with alterations in the immune microenvironment, macrophage‐associated markers were systematically examined. Flow cytometric analysis revealed that treatment with CD73^+^ADSCs significantly promoted macrophage polarization toward an M2 phenotype (Figure 5A). Next, western blot analysis revealed that LPS stimulation markedly upregulated the expression of inflammasome‐related proteins, including ASC, caspase‐1, and NLRP3. In contrast, CD73^+^ADSC treatment significantly reduced the levels of these proteins (Figure 5B, C and Figure S3). In parallel, ELISA results showed that LPS‐induced secretion of the inflammasome‐dependent cytokines IL‐1β and IL‐18 was significantly decreased after CD73^+^ADSC treatment. (Figure 5D).

**Figure 5:**
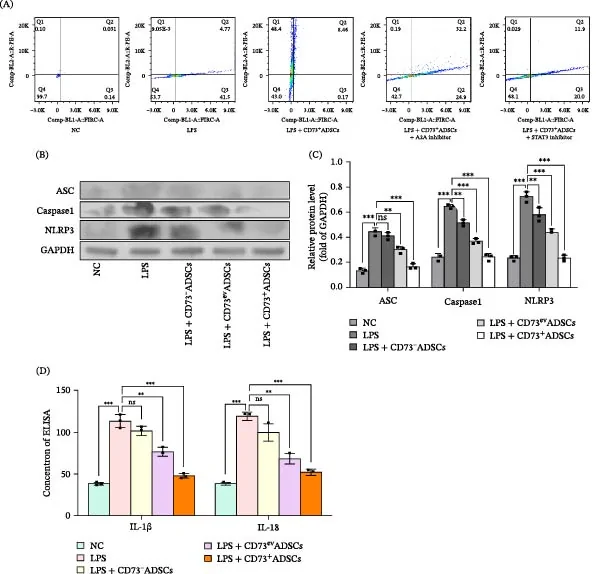
CD73‐overexpressing ADSCs regulate macrophage polarization toward the M2 and inhibit NLRP3 inflammasome activation. (A) Flow cytometry results showing macrophage polarization in each group. CD86 was detected using FITC‐conjugated antibody, and CD163 was detected using PE‐conjugated antibody. (B) Western blot and quantitative analysis of NLRP3, ASC, and caspase‐1 expression in vitro. (C) Quantitative analysis of Western blot. (D) ELISA analysis of IL‐1β and IL‐18 levels in each group in vitro. Statistical significance is indicated as  ^∗^ *p* < 0.05,  ^∗∗^ *p* < 0.01, and  ^∗∗∗^ *p* < 0.001. All experiments were independently performed in triplicate. The full‐length, uncropped Western blot images are provided in Figure S3. ELISA data are summarized in Tables S7 and S8.

Taken together, these results indicate that CD73^+^ADSCs remodel the inflammatory microenvironment by suppressing proinflammatory macrophage‐associated programs and promoting an immunoregulatory macrophage phenotype.

## 4. Discussion

NB remains difficult to treat because functional decompensation is driven not only by denervation but also by a self‐sustaining cycle of urothelial injury, inflammation, and maladaptive remodeling [17]. In this work, we show that CD73^+^ADSCs improve bladder functional readouts and tissue integrity in an NB model and, importantly, reshape inflammatory signaling in both the in vivo tissue and an in vitro LPS‐driven NB‐like microenvironment. Mechanistically, our findings suggest that CD73^+^ADSCs enhance adenosine generation and are associated with the activation of JAK1/STAT3 signaling. The partial reversal observed with an A2A antagonist supports the involvement of A2A‐related signaling, although additional genetic gain‐ and loss‐of‐function studies are required to establish direct causality. (Figure 6), driving macrophage M2 polarization, suppressing NF‐κB–NLRP3 inflammasome activation, reducing inflammation and apoptosis, and promoting repair of the NB microenvironment. A key advance of this study is the mechanistic coupling of a defined ectoenzyme enhancement to a coherent signaling cascade spanning adenosinergic signaling, JAK1/STAT3 activation, suppression of NF‐κB signaling, macrophage polarization, and inhibition of NLRP3 inflammasome activity. Rather than positioning CD73^+^ADSCs as a generic paracrine therapy, our data support the concept that augmenting the CD73–adenosine axis can amplify the ability of ADSCs to remain functionally active under inflammatory stress.

**Figure 6:**
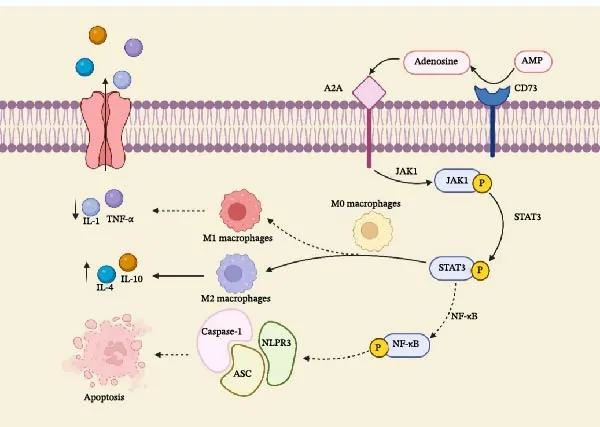
CD73^+^ADSCs enhance extracellular adenosine production through CD73‐mediated AMP hydrolysis, leading to activation of adenosine A2A receptor signaling. A2A engagement triggers the JAK1/STAT3 pathway, which promotes macrophage polarization toward an antiinflammatory M2 phenotype while suppressing proinflammatory M1 responses. Concurrently, STAT3 activation inhibits NF‐κB signaling and attenuates NLRP3 inflammasome assembly, resulting in reduced caspase‐1 activation, decreased IL‐1β and TNF‐α release, and increased IL‐4 and IL‐10 production. Through coordinated suppression of inflammation, inhibition of apoptosis, and reprogramming of the neurogenic bladder microenvironment, CD73^+^ADSCs facilitate tissue repair and functional recovery.

Most MSC‐based strategies for bladder dysfunction rely on unmodified cells, with efficacy frequently constrained by the hostile inflammatory niche [18]. Recent tissue‐engineered approaches underscore that improving delivery and retention can enhance functional outcomes in SCI‐associated bladder dysfunction [19], yet these approaches are not explicitly designed around a potential regulatory factor within the inflammatory microenvironment [20]. In contrast, our study focuses on a single CD73 that shifts extracellular purine metabolism toward adenosine production, thereby engaging endogenous antiinflammatory circuits. This design aligns with a broader trend in MSC biology: increasing evidence indicates that CD73 activity is not a passive surface marker but a determinant of immunomodulatory potency. Notably, previous researchers demonstrated that MSC exosomes can drive M2‐like macrophage polarization via implicating adenosine receptor‐dependent downstream signaling [21]. Our findings are consistent with this CD73‐centric framework but extend it in two important directions relevant to NB: we observe CD73‐associated suppression of NF‐κB signaling and inflammasome activity within an NB context, and we identify A2A–JAK1/STAT3 as a core intracellular axis linked to epithelial repair phenotypes.

A recurring question in adenosine biology is why A2A signaling couples to different intracellular modules across tissues [22]. MSC‐exosome literature often highlights AKT/ERK downstream of A2A/A2B in macrophages. In our system, the dominant repair‐associated node is JAK1/STAT3, supported by increased phosphorylation of JAK1/STAT3 and reversed by pharmacologic inhibition of A2A or STAT3. This apparent “pathway preference” can be interpreted by cell‐type and state dependence: macrophages frequently route adenosine signals through metabolic and MAPK hubs [23], whereas injured epithelial compartments commonly engage STAT3 as a canonical regeneration/survival module during acute stress [24]. This distinction is compatible with the broader MSC therapeutic landscape, where the same upstream cue can result in tissue‐specific repair programs.

A further nuance is that A2A signaling is increasingly recognized to have context‐dependent and even bidirectional roles in inflammation, depending on timing, ligand availability, and immune composition [7, 25]. Our data supports a model in which CD73^+^ADSCs create a localized injury‐conditioned adenosine tone that promotes a reparative STAT3 program while concurrently damping NF‐κB‐driven inflammation. This contextual model is also important for safety interpretation: the concern around STAT3 in urothelial carcinogenesis is largely linked to sustained [26], deregulated STAT3 activation, whereas our intervention appears to operate within a repair window and is coupled to an overall reduction in inflammatory stress, which is itself a driver of maladaptive proliferation.

Inflammation in NB is not limited to cytokine elevation; it involves coordinated activation of transcriptional programs and the innate immune machinery [27, 28]. Our data show that CD73^+^ADSCs reduce proinflammatory cytokines, suppress NF‐κB activation, and downregulate inflammasome components together with IL‐1β and IL‐18 output. This integrated suppression is conceptually consistent with recent mechanistic work in neuroinflammation [29], showing that interfering with A2A receptor–NLRP3 coupling can mitigate inflammasome assembly and downstream inflammation. While that work focuses on microglia, it strengthens the plausibility that A2A signaling can intersect with inflammasome control nodes. The specific molecular “wiring” may differ across tissues: CNS immune cells, bladder‐resident macrophages, and urothelial cells exhibit distinct receptor repertoires and metabolic baselines [30–32]. We propose that the consistency lies in a shared principle: adenosinergic signaling constrains inflammasome‐amplified inflammatory loops, whereas the identity of the intermediate kinases/transcriptional modules may be shaped by the dominant responding cell type in each microenvironment.

Macrophages play a pivotal role in bladder injury and repair, and their polarization state critically influences tissue outcomes [33]. Our data show that CD73^+^ADSCs promote macrophage polarization toward an M2‐like phenotype while concurrently suppressing activation of the NLRP3 inflammasome, as evidenced by reduced expression of NLRP3, ASC, and cleaved caspase‐1. Given the established contribution of NLRP3‐driven inflammation to bladder dysfunction across multiple etiologies [34], inhibition of inflammasome activation likely represents an important mechanism by which CD73^+^ADSCs limit inflammatory damage and facilitate tissue repair. Importantly, M2 polarization in this context was associated with improved bladder structure and function without evidence of excessive fibrosis. This suggests that macrophage reprogramming by CD73^+^ADSCs occurs within a coordinated signaling environment, characterized by reduced NF‐κB activation and controlled STAT3 signaling, favoring constructive remodeling rather than maladaptive scarring.

One risk in multiomics interpretation is overclaiming “regeneration” from pathway enrichment alone. In this study, the proteomic signatures provide directional reinforcement rather than standalone proof: CD73^+^ADSC exposure associates with downregulation of inflammatory regulators and enrichment changes in immune/metabolic pathways, alongside enrichment of DNA replication/cell cycle programs. Importantly, these proteomic patterns align with our targeted readouts, reduced NF‐κB/inflammasome activity, and increased repair‐associated signaling, supporting a coherent cross‐scale narrative.

From a translational perspective, our findings support CD73 as a rational target to enhance ADSC‐based therapy for NB. Augmenting CD73 activity may improve the efficacy of autologous ADSCs in inflammatory microenvironments without altering cell identity and suggests that mechanism‐based release criteria, such as adenosine‐generating capacity, could complement conventional phenotyping. In addition, CD73‐enriched extracellular vesicles or secretome‐based products may represent potential cell‐free alternatives, although their suitability for local bladder delivery requires further validation [35]. The partial dependence on the CD73–A2A axis further indicates opportunities for stage‐ or etiology‐specific combination strategies that reduce upstream inflammatory stress. Finally, biomarkers linked to inflammasome activity, NF‐κB signaling, and adenosinergic tone may enable patient stratification and response monitoring in the heterogeneous NB population.

Several limitations should be acknowledged. First, we used a single experimental NB paradigm, which may not capture the full etiologic heterogeneity of human NB. Second, although A2A inhibition supports involvement of the CD73–adenosine signaling axis, the relative contribution of other adenosine receptors was not examined, despite evidence for receptor‐ and context‐dependent immunomodulatory effects [36]. Third, our omics analyses were intended to inform mechanisms relevant to human disease, whereas the in vitro validation studies were conducted in rat‐derived cells. This cross‐species design is a limitation, although the major pathways identified by omics profiling were consistently confirmed in cellular experiments, supporting the robustness of the mechanistic conclusions. Future studies using single‐cell and spatial approaches will be needed to resolve cell‐type‐specific signaling and define the anatomical and cellular contexts in which CD73‐driven effects are most prominent. Finally, longer‐term studies are required to assess durability and safety, especially with respect to sustained STAT3 activation and the potential systemic immunomodulatory consequences of enhanced adenosine signaling.

## 5. Conclusion

Our findings suggest that CD73 overexpression may enhance the reparative and immunomodulatory properties of ADSCs in NB, potentially through regulation of adenosine signaling, JAK1/STAT3 activation, inflammatory responses, macrophage polarization, and inflammasome activity. These results provide preliminary mechanistic insight into the role of CD73 in the injured bladder microenvironment and support further investigation of CD73‐modified ADSCs as a potential therapeutic strategy for NB.

## Author Contributions

Guanqun Zhu and Guoyi Jiang conceived and designed the study, performed the main experiments, analyzed the data, and drafted the manuscript. Jiao Huang participated in the experimental design, data collection, and statistical analysis. Yuxuan He and Zongliang Zhang assisted with animal experiments, histological analysis, and data interpretation. Rui Zhang and Xinbao Yin contributed to the molecular biology experiments and figure preparation. Kai Zhao and Xiaokun Yang provided technical support and assisted in data validation and manuscript revision. Woong Jin Bae contributed to study supervision, critical revision of the manuscript, and provided important intellectual input. Ke Wang supervised the entire project, secured funding, finalized the study design, and critically revised the manuscript.

## Funding

This research was supported by a grant from the National Natural Science Foundation of China (Grant 82200759).

## Disclosure

All authors read and approved the final manuscript.

## Ethics Statement

The study protocol received approval from the Ethics Committee of the Affiliated Hospital of Qingdao University (Approval Number QYFYEC2023‐67; July 5, 2023). All animals were housed under standard laboratory conditions with free access to food and water. Every effort was made to minimize animal suffering and to reduce the number of animals used. All surgical procedures were performed under general anesthesia, and animals were humanely euthanized at the end of the experiments in accordance with ethical guidelines.

## Conflicts of Interest

The authors declare no conflicts of interest.

## Supporting Information

Additional supporting information can be found online in the Supporting Information section.

## Supporting information


**Supporting Information** The Supporting Information provides additional experimental data and methodological details supporting the results presented in the main text. Table S1 lists all primary antibodies, secondary antibodies, and key reagents used in this study. Figures S1–S3 show the full, uncropped western blot images corresponding to the blots presented in the main figures. Tables S2–S8 include raw ELISA data used for cytokine quantification. Figure S9 shows the original CCK‐8 assay data used for quantitative evaluation of cell proliferation.

## Data Availability

The data that support the findings of this study are available from the corresponding author upon reasonable request.
