# Supplementary material for: CD73‐Overexpressing ADSCs Promote Neurogenic Bladder Repair via A2A–JAK1/STAT3‐Mediated Modulation of Inflammation and Macrophage Polarization
Source: Stem Cells Int. 2026 Sep 29;2026:8564160. doi: 10.1155/sci/8564160 (PMC13624695; doi:10.1155/sci/8564160)
Supplement: Supplementary file 1 [file SCI-2026-8564160-s001.zip › Supplementary Materials.pdf]

Table S1.Primary antibodies and RNA sequences used in this study

Western blot	CD73 (diluted 1:1000, Affinity, Ontario, Canada),<br>A2AAR (diluted 1:1000, Abclonal , Wuhan, Hubei, China)<br>p-STAT3 (diluted 1:1000, Affinity, Ontario, Canada)<br>STAT3 (diluted 1:1000, Affinity, Ontario, Canada)<br>p-JAK1 (diluted 1:1000, Affinity, Ontario, Canada)<br>JAK1 (diluted 1:1000, Affinity, Ontario, Canada)<br>Cyclin D1(diluted 1:1000, Cell Signaling Technology, Danvers, MA, USA)<br>BCL-2(diluted 1:1000, Cell Signaling Technology, Danvers, MA, USA)<br>p-NFκB (diluted 1:1000, Cell Signaling Technology, Danvers, MA, USA)<br>NF-κB (diluted 1:1000, Cell Signaling Technology, Danvers, MA, USA)<br>ASC (diluted 1:1000, Affinity, Ontario, Canada), IKK (diluted 1:1000, Affinity, Ontario, Canada)<br>caspase1 (diluted 1:1000, Cell Signaling Technology, Danvers, MA, USA)<br>NLRP3 (diluted 1:1000, Cell Signaling Technology, Danvers, MA, USA)<br>GAPDH (diluted 1:1000, Abcam, Cambridge, UK)<br>β-actin (diluted 1:1000, Abcam, Cambridge, UK)
RNA	Nt5e(CD73)-rat-1277<br>(sense, 5’→3’)<br>GGACAGAGUGGUCCAAUUATT<br>(antisense, 5’→3’)<br>UAAUUGGACCACUCUGUCCTT<br><br>siRNA (Rat β-actin)<br>(sense, 5’→3’)<br>GUAAAGACCUCUAUGCCAATT<br>(antisense, 5’→3’)<br>UUGGCAUAGAGGUCUUUACTT
Immunofluorescence Staining and Histology	P-STAT3 (diluted 1:200, Santa Cruz Biotechnology, Santa Cruz, Dallas, USA),<br>P-JAK1(diluted 1:200, Abways, Shanghai, China)<br>BCL-1 (diluted 1:200, Servicebio, Wuhan, Hubei, China)<br>p-NFκB (diluted 1:1000, Cell Signaling Technology, Danvers, MA, USA)

Fig. S1. Full-length, uncropped Western blot images of A2A and CD73.

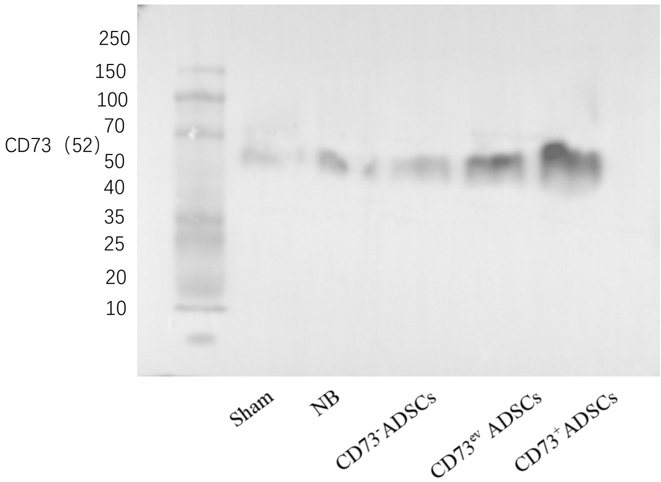

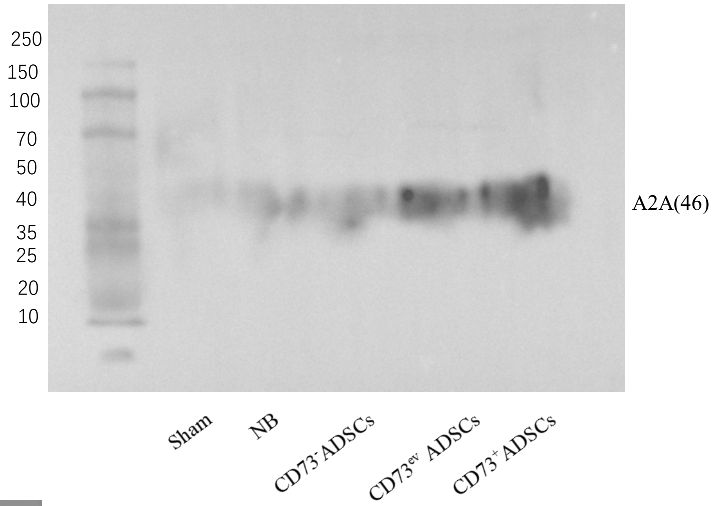

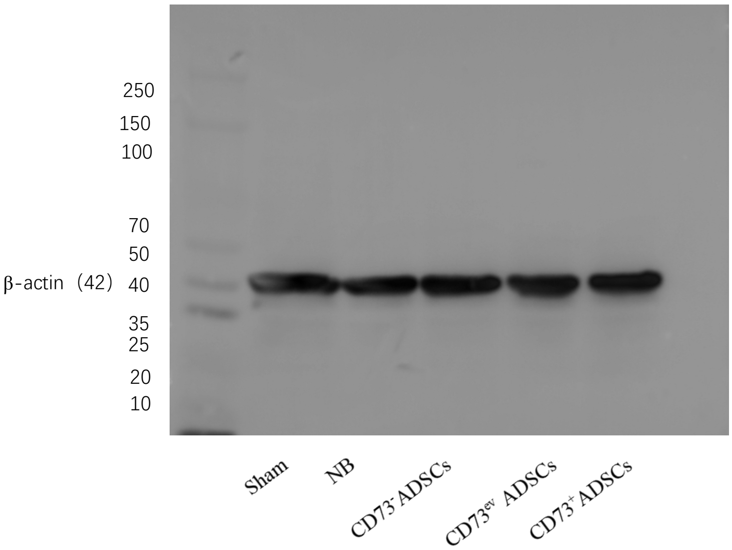

Fig. S2. Full-length, uncropped Western blot images of p-JAK1, JAK1, p-STAT3, STAT3, p-NF-κB, and NF-κB .

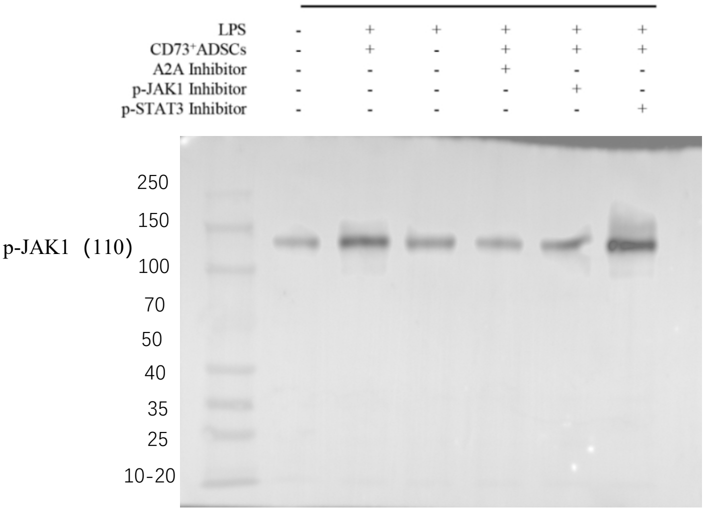

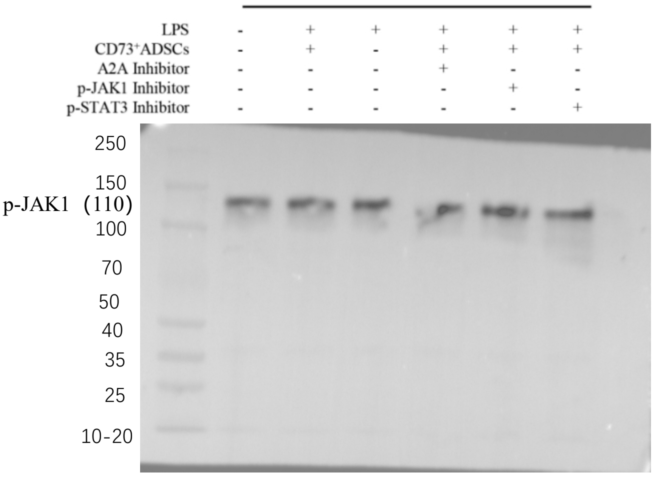

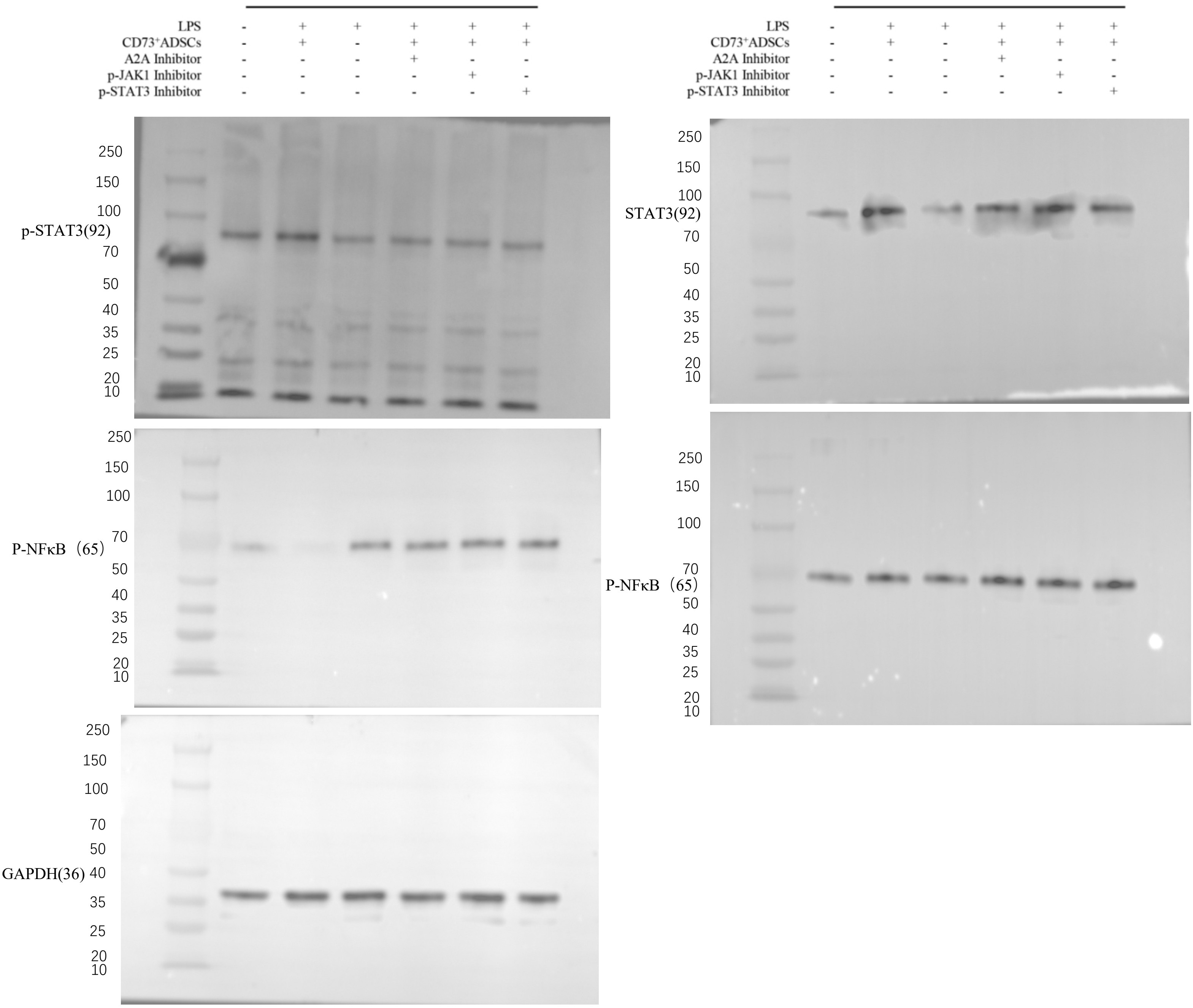

LPS - + + + + +
CD73⁺ADSCs - + - + + +
A2A Inhibitor - - - + - -
p-JAK1 Inhibitor - - - - + -
p-STAT3 Inhibitor - - - - - +
p-STAT3(92)
P-NFκB（65）
GAPDH(36)
STAT3(92)
250
150
100
70
50
40
35
25
20
10

Fig. S3.Full-length, uncropped Western blot images of NLRP3, ASC, caspase-1.

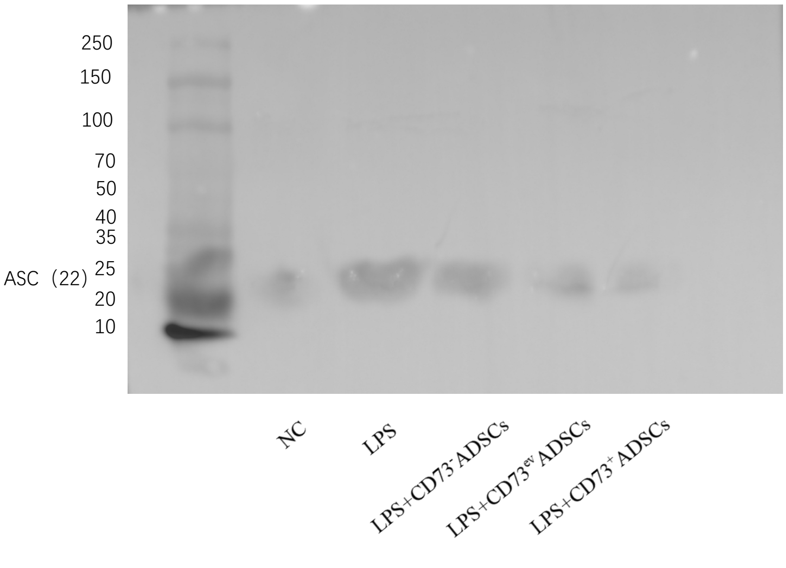

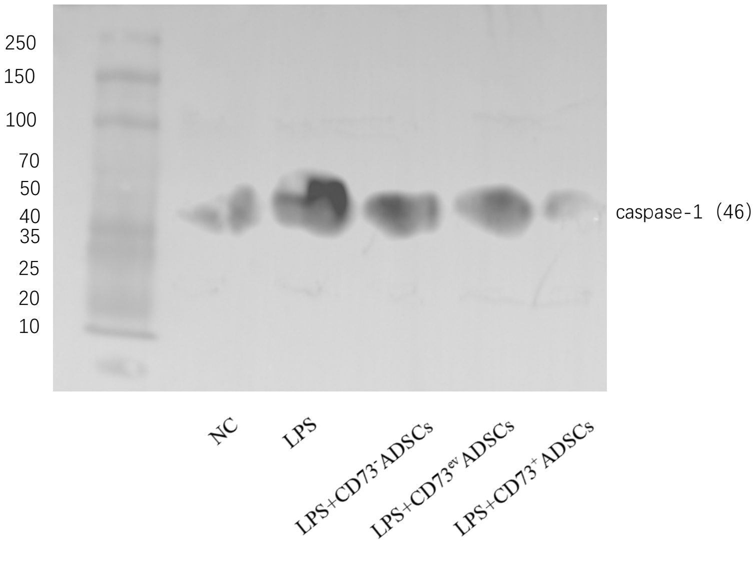

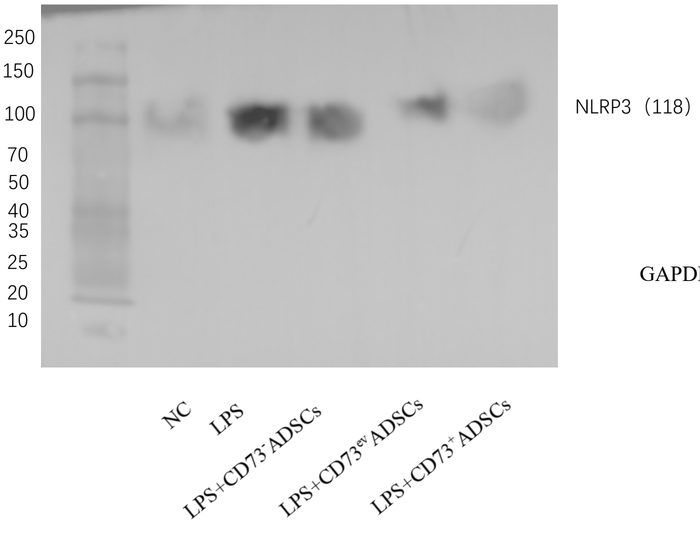

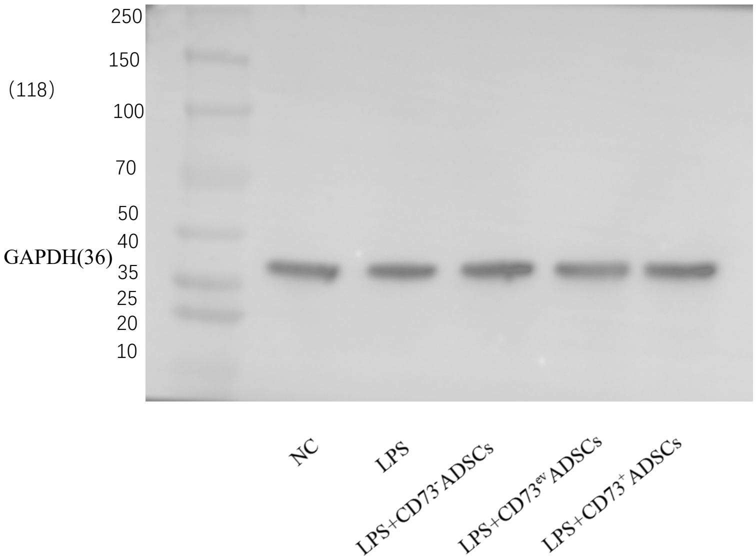

Table S2 ELISA data of IL-1β levels in each group.

IL-1β									
standard	OD			od			pg/ml		
0	0.0410		NC	0.0845	0.0863	0.0889	36.85	38.26	40.22
31.3	0.0771		LPS	0.1532	0.1438	0.1487	121.23	105.31	113.67
62.5	0.1185		LPS+CD73^{+}ADSCs	0.0973	0.1055	0.0957	46.53	52.73	45.28
125	0.1554		LPS+CD73+ADSCs^{+}A2AInhibitor	0.1303	0.1239	0.1261	82.42	71.63	75.41
250	0.3317								
500	0.5448								
1000	1.3461								
2000	2.56371								

Table S3 ELISA data of IL-6β levels in each group.

IL-6β									
standard	OD			od			pg/ml		
800	2.2471		NC	0.2419	0.2332	0.2505	36.26	34.26	37.84
400	1.4636		LPS	0.6137	0.5347	0.5717	132.53	112.23	121.72
200	0.8622		LPS+CD73+ADSCs	0.4249	0.4222	0.445	73.45	72.69	77.81
100	0.4524		LPS+CD73+ADSCs+A2AInhibitor	0.5118	0.4771	0.5081	104.53	95.73	103.28
50	0.2615								
25	0.1626								
12.5	0.1332								
0	0.1112								

Table S4 ELISA data of TNF-α levels in each group.

TNF-α									
standard	OD			od			pg/ml		
800	1.325		NC	0.1754	0.2060	0.1842	34.21	38.24	35.21
400	1.261		LPS	0.5021	0.5985	0.5596	89.23	101.37	95.83
200	0.863		LPS+CD73+ADSCs	0.3385	0.2882	0.3347	62.13	55.12	61.26
100	0.583		LPS+CD73+ADSCs+A2AInhibitor	0.4834	0.4573	0.4347	84.35	80.62	77.36
50	0.266								
25	0.125								
12.5	0.034								
0	0.017								

Table S5 ELISA data of IL-4 levels in each group.

IL-4								
standard	OD		od			pg/ml		
800	2.2361	NC	0.2351	0.2193	0.2747	37.42	33.57	45.22
400	1.2521	LPS	0.2125	0.2361	0.2531	32.33	38.43	41.82
200	0.8532	LPS+CD73+ADSCs	0.4577	0.4125	0.4297	82.42	71.63	75.41
100	0.5351	LPS+CD73+ADSCs+A2AInhibitor	0.3192	0.3439	0.2743	56.53	62.73	45.28
50	0.3662							
25	0.1721							
12.5	0.1128							
0	0.0415							

Table S6 ELISA data of IL-10 levels in each group.

IL-10								
standard	OD		od			pg/ml		
800	2.2641	NC	0.3010	0.3084	0.2731	47.62	49.17	42.35
400	1.2637	LPS	0.2743	0.2783	0.2712	42.62	43.17	41.75
200	0.6742	LPS+CD73+ADSCs	0.4647	0.4881	0.5081	98.33	104.27	94.76
100	0.4541	LPS+CD73+ADSCs+A2AInhibitor	0.3607	0.3684	0.3482	65.79	67.52	63.36
50	0.3132							
25	0.2128							
12.5	0.1262							
0	0.0415							

Table S7 ELISA data of IL-1β levels in each group.

IL-1β									
standard	OD		od			pg/ml			
0	0.0403	NC	0.0797	0.0826	0.0866	36.85	38.26	40.22
31.3	0.0782	LPS	0.1827	0.1639	0.1756	121.23	105.31	113.67
62.5	0.1265	LPS+CD73-ADSCs	0.1616	0.1567	0.1476	106.53	102.73	95.28
125	0.1834	LPS+CD73evADSCs	0.1348	0.1196	0.1258	82.42	71.63	75.41
250	0.3416	LPS+CD73+ADSCs	0.0955	0.1024	0.0925	47.21	50.67	45.23
500	0.5718							
1000	1.4466							
2000	2.5331							

Table S8 ELISA data of IL-8 levels in each group.

IL-18									
standard	OD		od			pg/ml			
0	0.0403	NC	0.0694	0.0718	0.075	36.85	38.26	40.22
31.3	0.0682	LPS	0.1831	0.1724	0.1827	122.33	113.43	121.82
62.5	0.1061	LPS+CD73-ADSCs	0.1414	0.1686	0.1457	92.42	111.63	95.41
125	0.1794	LPS+CD73evADSCs	0.1112	0.106	0.1247	66.53	62.73	75.28
250	0.3117	LPS+CD73+ADSCs	0.0914	0.0838	0.094	53.25	48.2	54.97
500	0.5416							
1000	1.1476							
2000	2.0351							

Table S9 CCK-8 data of each group.

CCK8(OD)															
DAY	NC			LPS			LPS+CD73-ADSCs			LPS+CD73EVADSCs			LPS+CD73+ADSCs		
1	0.2725	0.2463	0.2784	0.2642	0.2701	0.2519	0.2771	0.2635	0.2426	0.2593	0.2753	0.2576	0.2402	0.2739	0.2687
2	0.2783	0.2812	0.2796	0.2764	0.2825	0.2996	0.3003	0.3005	0.2749	0.2995	0.3398	0.3441	0.3412	0.3365	0.3424
3	0.3675	0.3321	0.3298	0.3546	0.391	0.4373	0.3525	0.4491	0.4056	0.4115	0.5438	0.5064	0.5392	0.5516	0.518
4	0.4015	0.4589	0.4263	0.4237	0.4196	0.5518	0.5184	0.4996	0.5152	0.485	0.7189	0.7025	0.6753	0.7124	0.7509
5	0.5512	0.4928	0.5843	0.5937	0.588	0.7185	0.6653	0.7261	0.7098	0.6803	0.9387	0.9624	0.9453	0.9316	0.962
