# Supplementary material for: CD73‐Overexpressing ADSCs Promote Neurogenic Bladder Repair via A2A–JAK1/STAT3‐Mediated Modulation of Inflammation and Macrophage Polarization
Source: Stem Cells Int. 2026 Sep 29;2026:8564160. doi: 10.1155/sci/8564160 (PMC13624695; doi:10.1155/sci/8564160)

Fig. 1

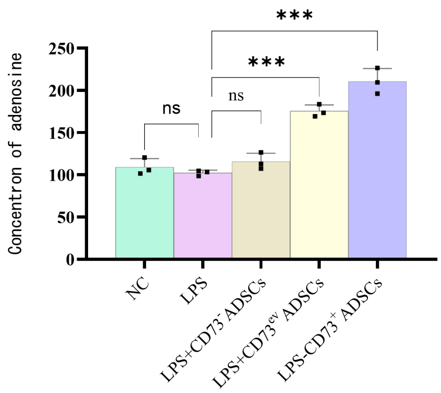

Adenosine									
standard	OD			od			pg/ml		
0	0.0453		NC	0.1813	0.1641	0.1710	105.67	120.47	101.55
31.3	0.0862		LPS	0.1659	0.1669	0.1647	98.67	103.54	104.67
62.5	0.1285		LPS+CD73-	0.1694	0.1877	0.1772	112.95	107.43	126.53
125	0.1854		LPS+CD73ev	0.2576	0.2728	0.2606	169.25	173.41	183.64
250	0.3717		LPS+CD73+	0.2916	0.3366	0.3132	209.56	196.24	226.47
500	0.6628								
1000	1.2361								
2000	2.0637								

Fig. 2

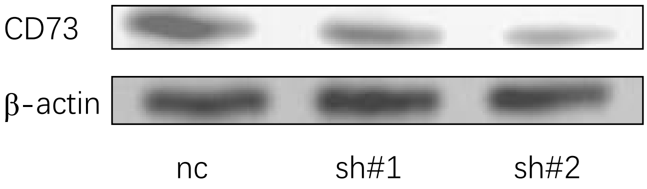

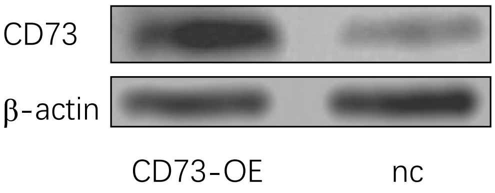

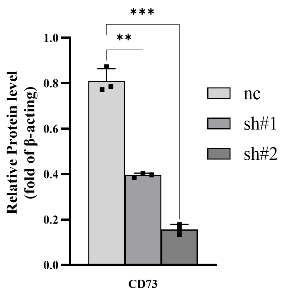

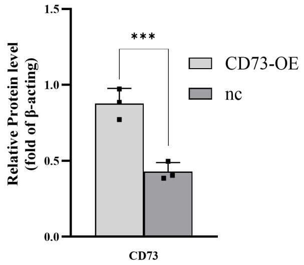

Western blot validation of CD73 knockdown

Western blot validation of CD73 overexpression

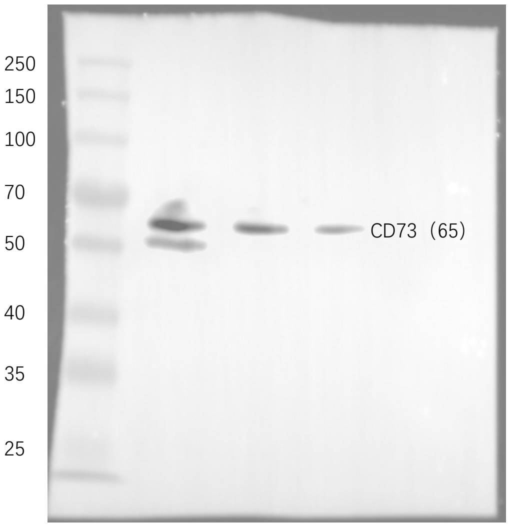

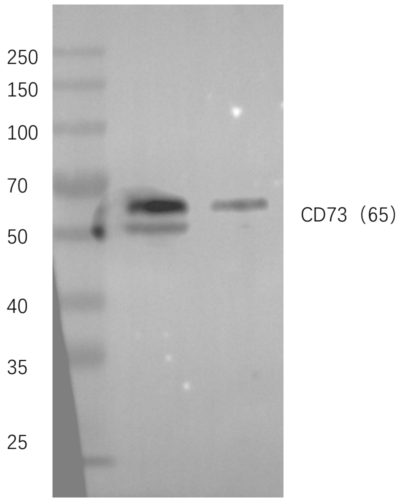

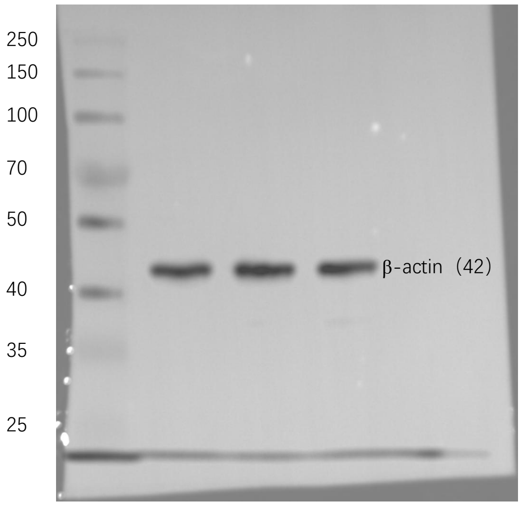

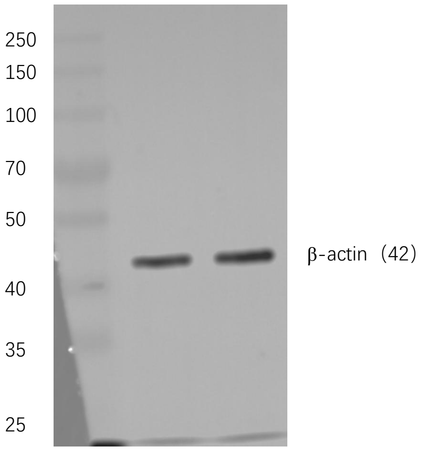

Supplement: Supplementary file 1 [file SCI-2026-8564160-s001.zip › Supplementary Materials 2.pdf]
